# Spatial Distribution and Associated Factors of Institutional Delivery among Reproductive-Age Women in Ethiopia: The Case of Ethiopia Demographic and Health Survey

**DOI:** 10.1155/2022/4480568

**Published:** 2022-06-25

**Authors:** Daniel Sisay, Helen Ali Ewune, Temesgen Muche, Wondwosen Molla

**Affiliations:** ^1^School of Public Health, College of Health Science and Medicine, Dilla University, Dilla, P.O. Box 412, Ethiopia; ^2^Department of Midwifery, College of Health Science and Medicine, Dilla University, Dilla, P.O. Box 412, Ethiopia

## Abstract

**Background:**

Maternal mortality is unacceptably high. About 295,000 women died during and following pregnancy and childbirth in 2017. The vast majority of these deaths (94%) occurred in low-resource settings, and most could have been prevented.

**Methods:**

This research is based on a cross-sectional study using 2016 EDHS data. The analysis included 7,590 women who had given birth in the five years prior to the survey. Clusters with high and low hot spots with institutional delivery were found using SatScan spatial statistical analysis. A multilevel multivariable mixed-effect logistic regression was utilized to discover characteristics associated with institutional delivery.

**Result:**

In this study, 33.25% of women who gave birth in the last 5 years preceding the survey delivered their babies at health institutions. The finding also indicated that the spatial distribution of institutional delivery was nonrandom in the country. Variables achieving statically significant association with utilization of institutional delivery were as follows: at the individual level, richness (AOR = 2.18, 95%CI: 1.39–3.41), higher education (AOR = 3.89, 95%CI: 1.51–10.01), a number of antenatal care visits of four and above (AOR = 6.57, 95%CI: 4.83–8.94), and parity of more than two children (AOR = 0.48, 95%CI: 0.34–0.68); at the community level, higher education (AOR = 1.70, 95%CI: 1.22–2.36) and urban residence (AOR = 5.30, 95%CI: 3.10–9.06) were variables that had achieved statically significant association for utilization of institutional delivery.

**Conclusions:**

This study identified a spatial cluster of institutional delivery with the Somali and Afar region having low utilization rates and Addis Ababa and Tigray regions having the highest utilization rates. The significant individual factors associated with institution delivery were woman antenatal care visits, household wealth index, maternal education, and parity, and the significant community ones were region, place of residence, and educational status. Therefore, to maximize health facility delivery in Ethiopia, the predictors of institutional delivery identified in this study should be given more attention by governmental and nongovernmental stakeholders.

## 1. Background

Maternal mortality is one of the major challenges in Africa, and its disparity between developing and developed countries is very high. The maternal mortality rate in developing regions is 15 times higher than that in developed regions. Developing countries including Ethiopia have the highest maternal mortality rate in the world with an average of 500 maternal deaths per 100,000 live births (Central Statistical Agency (CSA)) [1].

Maternal mortality is unacceptably high. About 295,000 women died during and following pregnancy and childbirth in 2017. The vast majority of these deaths (94%) occurred in low-resource settings, and most could have been prevented [2].

Almost three-fourths of deliveries were assisted by skilled professionals globally, and only half, less than half, and one-fourth of deliveries in Africa, sub-Saharan Africa, and Ethiopia were assisted by a skilled health professional in 2015 [3].

Regardless of the Ethiopian government's efforts to increase health service facilities and promote institution-based delivery providing in the country, a predicted percentage of births of 85% still take place at home [3, 4].

Maternal death is one of the causes of personal and social distress in families, because women have major tasks in most family problems, such as raising children, and have a major role in society [5].

The MMR was 673 per 100,000 live births in 2005 [6], 676 per 100,000 live births in 2011 [7], and 412 per 100,000 live births in 2016.

To ensure that every baby is delivered in a healthcare facility with the assistance of a professional healthcare attendant is one essential method for minimizing maternal morbidity and mortality. To prevent maternal mortality, the most effective method for low-income nations is to promote birthing at healthcare institutions with referral capability, as timely management and treatment can mean the difference between life and death [8].

In Ethiopia, just like most developing countries, only 15% of births are delivered at a health facility despite more than 40% of pregnant women having at least one antenatal care visit during pregnancy [9].

Furthermore, according to the 2005 EDHS, the majority of home births occur in unsanitary conditions, whereas just 6% occur in health institutions and are helped by skilled staff [10]. Formerly, there were studies conducted to identify the determinants of institutional delivery among women of reproductive age [11]. Despite the fact that institutional delivery is low in Ethiopia, there have been few studies on the determinants of spatial distribution that have not taken into account the hierarchical and cluster nature of institutional delivery. Identifying the factors and distribution of institutional delivery in space is very crucial for increasing delivery of mothers at healthcare facilities and to provide geographic-based evidence for decision-makers to formulate appropriate plans. Therefore, the current study objective is to explore the spatial distribution and associated factors of institutional delivery in Ethiopia among reproductive-age women, using advanced level modeling.

## 2. Methods

### 2.1. Study Design and Period

Secondary data analysis was carried out using the EDHS data of 2016, from January 18 to June 27, 2016.

### 2.2. Study Area

The study was conducted in Ethiopia latitude and longitude of 8°00 N and 38°00 E, situated at the eastern Horn of Africa. The country covers 1.1 million square kilometers and has a great geographical diversity, which ranges from 4,550 meters above sea level down to the Afar depression to 110 meters below sea level. There are nine regional states and two city administrations subdivided into 68 zones, 817 districts, and 16,253 kebeles (lowest local administrative units in the administrative structure of the country). The analysis included 7,590 reproductive-age women who had given birth in the five years prior to the survey.

### 2.3. Source Population

Source population consisted of all reproductive women aged 15–49 years in Ethiopia who gave birth in the last 5 years preceding the survey.

#### 2.3.1. Study Population

Study population consisted of all women aged 15 to 49 years in the selected enumeration areas who gave at least one birth in the last 5 years preceding the survey.

### 2.4. Inclusion and Exclusion Criteria

#### 2.4.1. Inclusion Criteria

All women who gave birth in the past 5 years before the survey in the selected enumeration areas were included.

#### 2.4.2. Exclusion Criteria

Women with unknown places of delivery out of the geographical positioning system were excluded.

### 2.5. Sample Size Estimation

In the 2016 Ethiopia Demographic and Health Survey (EDHS), 15,683 women aged 15 to 49 were interviewed; 7,590 women had given birth in the five years prior to the interview.

### 2.6. Sampling Procedures

Administratively, regions in Ethiopia are divided into zones, and zones are divided into administrative units called woredas. Each woreda is further subdivided into the lowest administrative units, called kebeles. During the 2007 census, each kebele was subdivided into census enumeration areas (EAs), which were convenient for the implementation of the census. A stratified two-stage cluster sampling procedure was employed where EA is the sampling unit for the first stage and households for the second stage. In 2016 EDHS, a total of 645 EAs (202 in urban areas and 443 in rural areas) were selected with probability proportional to EA size (based on the 2007 housing and population census) and with independent selection in each sampling stratum. There were 7,590 women among the 18,060 households.

### 2.7. Study Variables

#### 2.7.1. Dependent Variable

The dependent variable is institutional delivery (yes or no).

#### 2.7.2. Independent Variables

(1) Individual-Level Institutional Variables

Sociodemographic factors
    Age at first birth
    Husband's education
    Religion
    Residence
    Maternal education
    Wealth index
    Maternal age
    Marital status
Obstetrics factors

  Antenatal care service
  Parity
  Birth order

(2) Community-level institutional variables

  Region (pastoralist, agrarian, and city)
  Place of residence
  Community-level poverty
   Community-level education

### 2.8. Operational Definition

#### 2.8.1. Institutional Delivery

The major response variable in this study was institutional delivery, whether women have had a live birth recently or not. The EDHS place of delivery was measured by questioning women, “Where did you give birth?” The answer was assigned the code “1” if the mother gave birth in a hospital (governmental or nongovernmental health center) and “0” if the woman gave birth somewhere else.

#### 2.8.2. Antenatal Care Utilization

At least one visit was made to ANC.

#### 2.8.3. Community

According to the EDHS data, the primary sample unit is defined. Individual-level data were aggregated to produce community-level variables, which were calculated using averages of the proportion of women in each category of a given variable based on national median values. The values have been gathered and sorted into groupings [12].

#### 2.8.4. Community Poverty

The proportion of women in the community who live in the lower (poor) and higher (rich) quintiles of the wealth index is classified as high (proportion of women greater than the median national value) or low (proportion of women less than the median national value).

#### 2.8.5. Community Education

The proportion of women in the community who primary and secondary, categorized as high (proportion of women greater than median national value) whereas low (proportion of women below-median national value).

#### 2.8.6. Region

The 11 regions of Ethiopia, which are delineated for administrative purposes, were categorized into three contextual regions: pastoralist (Afar, Gambella, Benishangul-Gumuz, and Somali); agrarian (Amhara, Tigray, Oromia, and SNNPR); and city (AA, Dire Dawa, and Harari) defined based on the living conditions of their population [13].

### 2.9. Data Collection and Quality Assurance

#### 2.9.1. Data Source

Data were obtained from the nationally representative 2016 Ethiopia Demographic and Health Survey (EDHS) which used a two-stage cluster sampling design with rural-urban regions as strata. Approval letter for the use of this data was gained from the MEASURE DHS.

### 2.10. Data Analysis

First, data were extracted and cleaned by Stata version 14.1. A multilevel logistic regression analysis technique was employed in this study to account for the hierarchal structure of the DHS data and the binary response of the outcome variable. Bivariate multilevel logistic regression analysis was performed to estimate the crude odds ratios at 95% confidence interval, and those variables which were statistically significant were considered in the multivariate analysis. Finally, multivariate multilevel logistic regression analysis was performed to estimate the adjusted odds ratios and to estimate the extent of random variations between communities. In the multilevel models, the fixed effects (measures of association) estimate the association between the likelihood of institutional delivery and the individual- and community-level factors and were expressed as odds ratio with their 95% confidence intervals. The random effects are the measures of variation in institutional delivery across communities expressed as intracluster correlation coefficient (ICC) and proportional change in variance (PCV).

Model comparison was conducted by using the log-likelihood ratio test, and the model with the maximum LLR was selected as a better-fitted model. A geographical information system (ArcGIS version 10.6) and spatial SatScan were used to analyze spatial data.

#### 2.10.1. Spatial Autocorrelation Analysis

The spatial autocorrelation (Global Moran's I) statistic measures were used to determine whether the institutional delivery patterns in the study area are dispersed, clustered, or randomly distributed. When Moran's I values near 1 indicate that institutional delivery is randomly distributed, whereas Moran's I values near +1 indicate that institutional delivery is clustered distributed. A statistically significant Moran's I (*p* < 0.05) leads to rejection of the null hypothesis and indicates the presence of spatial autocorrelation. Local Moran's I will be used to investigate the local level cluster locations of institutional delivery. Local Moran's I measures whether there were positively correlated (high-high and low-low) clusters or negatively correlated (high-low and low-high) clusters of high values (high-high) or low values (low-low). It also measures an outlier in which high value is surrounded primarily by low values, and an outlier in which a low value is surrounded primarily by high values. Value for “*I*” indicated that a case is surrounded by cases with dissimilar values; this case is an outlier.

### 2.11. Hot Spot Analysis (Getis-Ord Gi^*∗*^ Statistic)

Getis-Ord Gi^*∗*^ statistics were computed to measure how spatial autocorrelation varies over the study location by calculating Gi^*∗*^ statistic for each area. The *z*-score is computed to determine the statistical significance of clustering, and the *p*-value computed for the significance. The *p*-value associated with a 95% confidence level is 0.05. If the *z*-score is between −1.96 and +1.96, the *p*-value will be larger than 0.05, and the null hypothesis cannot be rejected; the pattern exhibited could very likely be the result of random spatial processes. If the *z*-score falls outside the range, the observed spatial pattern is probably too unusual to be the result of random chance, and the *p*-value will be too small to reflect this. In this case, it is possible to reject the null hypothesis and proceed with figuring out what might be causing the statistically significant spatial pattern in the data. Statistical output with high Gi^*∗*^ indicates “hot spot” whereas low Gi^*∗*^ means a cold spot.

This study identified a spatial cluster of institutional delivery, with Somali and Afar region having low utilization rates and Addis Ababa and Tigray regions having the highest utilization rates.

#### 2.11.1. Incremental Analysis

Incremental spatial autocorrelation measures spatial autocorrelation for a series of distances and optionally creates a line graph of those distances and their corresponding *z*-scores. Statistically significant peak *z*-scores indicate distances where spatial processes promoting clustering are most pronounced. These peak distances are often appropriate values for tools with a Distance Band or Distance Radius parameter.

#### 2.11.2. Incremental Spatial Autocorrelation

The incremental spatial autocorrelation of institutional delivery utilization showed that the maximum peak, where the spatial clustering is highly significant at a distance of 151,366.65 meters, has a corresponding *z*-score of 25.53 (*p*-value <0.01).

### 2.12. Ethical Consideration

Ethical clearance was obtained from the Institutional Review Board (IRB) of the Institute of Public Health, College of Medicine and Health Sciences, University of Gondar. The survey data was received from the MEASURE DHS International Program which authorized the datasets. All the data used in this study are publicly available, aggregated secondary data with no personal identifying information that can be linked to particular individuals, communities, or study participants. Confidentiality of data was maintained anonymously.

## 3. Results

### 3.1. Sociodemographic Characteristics of Study Participants

In this study, a total of 7,590 women having their most recent birth in the five years preceding the 2016 EDHS survey were included in the analysis. Thirty-three percent of live births in the 5 years before the survey were delivered in a health facility. About one-fourth (24%) of participants were 15–24 years old, almost ninety percent (87%; 6,620) were rural residents, and the majority of women who participated in the survey were Orthodox Christians. Regarding the educational status of the participants, 4,791 (63%) have no education, and 1,654 (22%) were in the poorer wealth quintile (see Table 1).

### 3.2. Prevalence of Institutional Delivery across Regions

**Figure 1:**
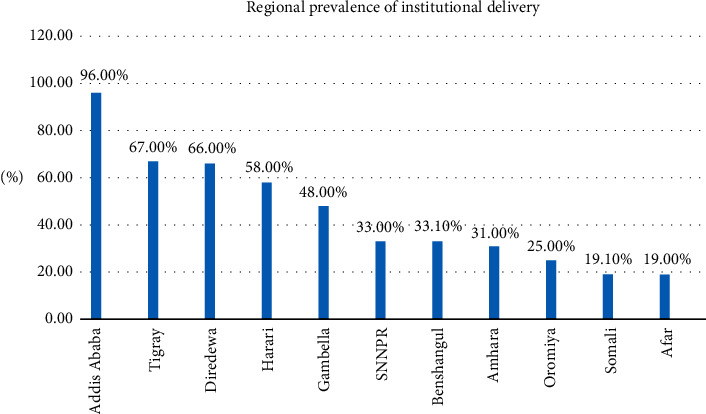
Regional prevalence of institutional delivery (source: shape file from Central Statistical Agency, 2016).

The prevalence of institutional delivery utilization varies across the regions of the country. The highest and the lowest prevalence were observed in Addis Ababa (96%) and Afar (19.0%) regions, respectively (Figure 1).

### 3.3. Spatial Analysis of the Geographic Information System

#### 3.3.1. Incremental Analysis

Incremental spatial autocorrelation measures spatial autocorrelation for a series of distances and optionally creates a line graph of those distances and their corresponding *z*-scores. Statistically significant peak *z*-scores indicate distances where spatial processes promoting clustering are most pronounced. These peak distances are often appropriate values for tools with a Distance Band or Distance Radius parameter.

#### 3.3.2. Spatial Autocorrelation Report

**Figure 2:**
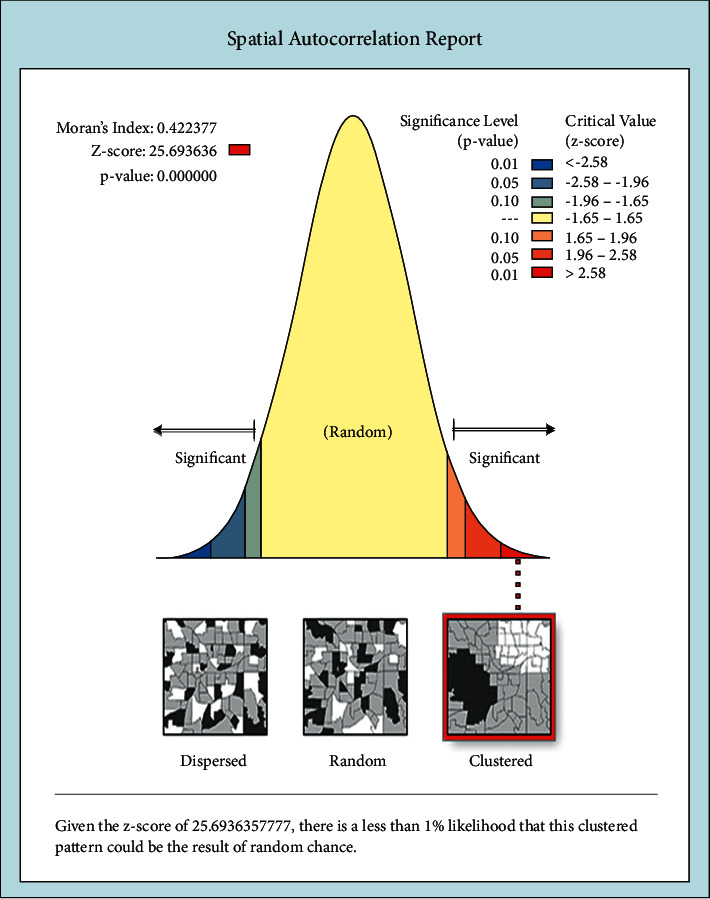
Spatial autocorrelation for distribution of institutional delivery in Ethiopia, 2016 (source: shape file from CSA, 2013, done using ArcGIS version 10.6).

Spatial autocorrelation in GIS helps understand the degree to which one object is similar to other nearby objects and measures how much close objects are comparable with other close objects. Analysis of institutional delivery spatial autocorrelation by Global Moran's I, with values of dispersed (Moran's I close to 1), random (0), or clustered (Moran's I close to +1). Then, the Global Moran's I values in our study were 0.42 (*P*-value 0.001), indicating that there was significant clustering of institutional delivery in the study area. The clustered patterns on the right show that more distribution occurred in the study area. The bright red and blue colors (to the end tails) indicate an increased significance level (Figure 2).

### 3.4. Hot Spot Analysis of Institutional Delivery across Regions of Ethiopia

**Figure 3:**
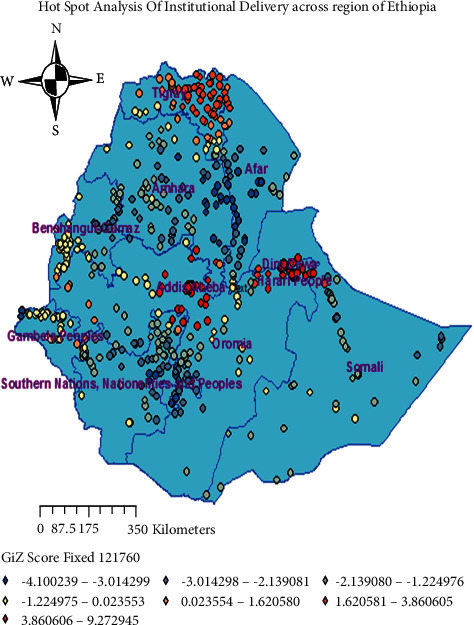
Hot spot analysis of institutional delivery in Ethiopia, EDHS 2016 (source: shape file from CSA, 2013, done using ArcGIS version 10.6).

The red color indicates significant areas (clusters of institutional delivery), whereas blue color shows significant home delivery areas based on the hot spot and cold spot analysis. When the *z*-score increases in both directions (+/), its significance level also increases. Positive *z*-score shows place of high utilization of institutional delivery, and negative *z*-score shows a place of low institutional delivery (risk of home delivery) across regions such as Afar, Somali, Benishangul-Gumuz, and west Oromia (Figure 3).

#### 3.4.1. Cluster and Outlier of Institutional Delivery across Regions

**Figure 4:**
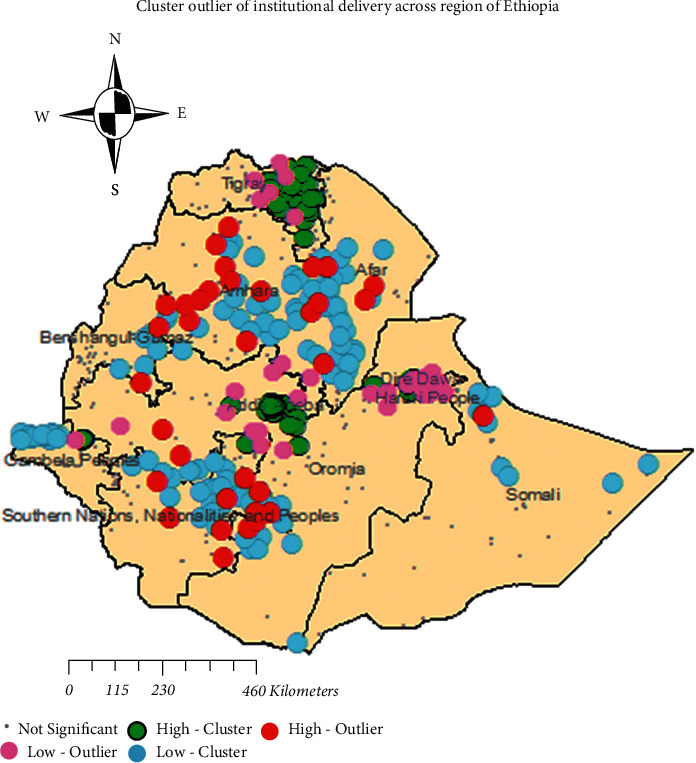
Cluster and outlier of institutional delivery in Ethiopia, 2016 (source: shape file from CSA, 2013, done using ArcGIS version 10.6).

  High-Cluster indicated that high rates of institutional delivery women's surrounded by similar characteristics, found regions of Addis Ababa, East Tigray, Dire Dawa, and Harari
  High-cluster indicated high rates of institutional delivery women, which is found in the Amhara and West Oromia regions surrounded by low rates of institutional delivery women.
  Low-Outlier indicated that low distribution of institutional delivery surrounded by the high distribution of institutional delivery found regions of Central Tigray, Dire Dawa, and Oromia
  Low-Cluster indicated that low distribution of institutional delivery surrounded by similar characteristics. Found regions of Afar, Somali, Central Amhara, and Benishangul-Gumuz (Figure 4).

#### 3.4.2. Spatial Interpolation across the Regions of Ethiopia, EDHS 2016

**Figure 5:**
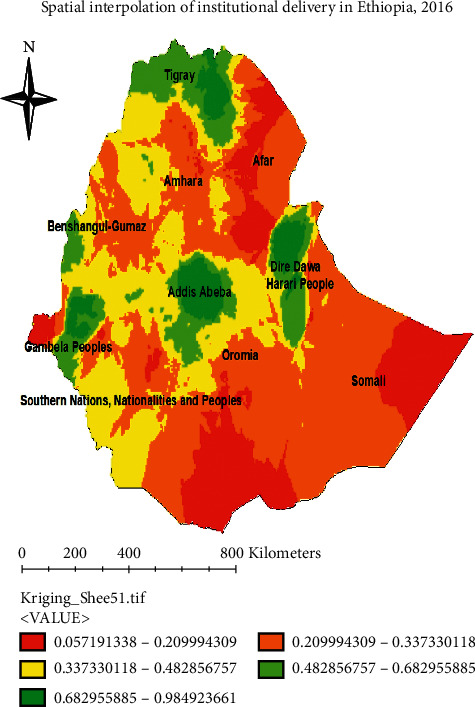
Spatial interpolation of institutional delivery areas across regions in Ethiopia, 2016 (source: shape file from CSA, 2013, done using ArcGIS version 10.6).

The map is made up of continuous images interpolated using the Kriging interpolation method, which predicts unknown values at other locations. The red color denoted a high likelihood of institutional delivery utilization. Addis Abeba, Tigray, Dire Dawa, Harari, and Gambella regions were found. Green color predicted a low proportion of institutional delivery utilization. The regions of central and northern Afar, eastern Somalia, Benishangul-Gumuz, and the eastern part of Oromia were found (Figure 5).

### 3.5. Determinants of Institutional Delivery

#### 3.5.1. Effect of Individual Women Characteristics on the Place of Delivery

Women who had primary, secondary, and higher education were, respectively, 1.44 times (AOR (95% CI) = 1.40 (1.11–1.77)), 3.25 times (AOR (95% CI) = 3.25 (2.03–5.19)), and 3.98 times (AOR (95% CI) = 3.98 (1.51–10.01)) more likely to give birth at a health facility as compared to those with no education.

Women who are poorer and richest were, respectively, 1.46 times (AOR (95% CI) = 1.46 (1.03–2.09)) and 2.18 times (AOR (95% CI) = 2.18 (1.39–3.41)) more likely to give birth at a health facility than women who are poorest.

Women who had more than one and more than four antenatal care visits were, respectively, 3.75 times (AOR (95% CI) = 3.75 (2.76–5.10)) and 6.57 times (AOR (95% CI) = 6.57 (4.83–8.94)) more likely to give birth in health institutions than women who did not have any antenatal care visits.

Regarding parity, women who had 2–4 children (52%) (AOR (95% CI) = 0.48 (0.34–0.68)) were less likely to deliver their babies at a health facility as compared to women who had one child. The study aimed to show if the characteristics of the clusters in which women lived would have an effect on their decision about the place of delivery. As for the place of residence, urban clusters had more odds of having a 5.30 times higher proportion of women giving birth at institutions (AOR (95% CI) = 5.30 (3.10–9.06)) in the community. Chance of institutional delivery in communities with a high proportion of educated women was 70% higher (AOR = 1.70; 95% CI = 1.70 (1.22–2.36)) as compared to communities with a low proportion of educated women.

The odds of health facility delivery among women living in pastoralist community is reduced by 70% (AOR = 0.30; 95% CI = 0.30 (0.19–0.48)), and similarly women living agrarian community had 55% (AOR = 0.45; 95% CI = 0.45 (0.30–0.69)) reduction in delivery at a health facility as compared with women in city communities (see Table 2).

#### 3.5.2. Random Effect and Model Comparison Parameters

In the null model, about 63% of the total variation in institutional delivery occurred at the community level and is attributable to the community-level factors. The highest MOR value (9.45) in the null model revealed there was a variation of institutional delivery between clusters. Furthermore, the highest (82.63%) PCV in the final model (Model 4) indicates that 82.63% of the variation in institutional delivery across communities was explained by both individual- and community-level factors. The model fitness was checked using the log-likelihood ratio, and the model with the lowest log-likelihood ratio (Model 4) was the best-fitted model (see Tables 3 and 4).

## 4. Discussion

This study aimed to explore the spatial distribution and associated factors of institutional delivery among women aged 15–49 years: the case of EDHS 2016 data. In this study, the prevalence of institutional delivery utilization was 33.25%. This result is lower than the results from India (84.9%) [14] and Tanzania (74.5%) [15] but higher than findings from the southeast part of Ethiopia (12.3%) [16] and Northwest Ethiopia (12.1%) [17]. Our findings show that the proportion of institutional delivery varies across the regions of the country. The highest and the lowest prevalence were observed in Addis Ababa (96%) and Afar (19.0%) regions, respectively. The lowest rate of institutional delivery was due to a lack of education and access to a maternal health delivery facility.

The geographical information system (GIS) analysis indicates that the spatial distribution of institutional delivery was nonrandom in the country. The Global Moran's I values of our findings are 0.42 (*p*-value <0.001) indicating that there was statistically significant clustering of institutional delivery in the study area. And cold spot also shows areas of low institutional delivery were found in Afar, Somali, and Benishangul-Gumuz regions. In addition, interpolation predicted that regions with a low proportion of institutional delivery are central and northern part of Afar, eastern Somali, and Benishangul-Gumuz and eastern part of Oromia. Generally, the above GIS spatial report shows that regions with low institutional delivery are Afar, Somali, Benishangul-Gumuz, and eastern part of Oromia. This is due to a lack of access to media exposure and transport system in addition to having poor infrastructure for the utilization of institutional delivery service.

Regarding the determinants of institutional delivery, the wealth index is a significant predictor of institutional delivery. The odds of giving birth at health institutions among mothers who are poorer and richest were 1.46 and 2.18 times, respectively, higher as compared to the poorest ones. This result is similar to those of the studies done in Bangladesh, Malawi Jacob, and Tigray (northern part of Ethiopia) [18–20].

This might be related to the costs needed to access healthcare services. Women's education is positively associated with institutional delivery in Ethiopia. The odds of giving birth at health institutions among mothers who had primary education, secondary education, and higher educational level were 1.40, 3.25, and 3.9 times higher as compared to uneducated women. This result is also similar to those of the studies done in Ethiopia in Bahir Dar city, in Bangladesh, and Pakistan. This might be due to educated women's awareness about the risk of home delivery and the importance of maternal health services [21–23].

The number of antenatal care visits is positively associated with health facility delivery. Mothers who had 1–3 and 4 and above ANC visits were 3.75 and 6.57 times more likely to deliver at a health facility, respectively, as compared to mothers with no ANC visits. This finding is consistent with the studies done in Northwest Ethiopia, Somali regional states, and India. The reason might be that those women attending antenatal care will be counseled about the importance of institutional delivery and birth preparedness plan [24–26].

Parity was another important predictor of institutional delivery. The odds of delivery at a health facility among mothers who had a number of living children of 2–4 were reduced by 52% as compared to those who had only one child. This finding is consistent with the study conducted in Pakistan and Rural Tanzania. The reason is that women who deliver their first child without any problem might think that they will not face difficulty in delivering their second baby and they may not also find a person who cares for their first child at home while they are at the health institution for delivery [21, 27–36].

Communities with a high proportion of educated women had a 70% higher chance of institutional delivery as compared to communities with a low proportion of educated women. This study is consistent with a study done in six African countries [37].

The reason might be that the community factor of education could influence women's overall empowerment, enhancing their ability to access information and easily absorb health messages through the media and health professionals. These could collectively influence mothers' awareness of the importance of seeking better medical services, including delivery in health facilities.

The odds of health facility delivery among women living in the pastoralist community are reduced by 70%, and similarly, women living in the agrarian community had a 55% reduction in delivery at a health facility as compared with women in city communities. This is due to the fact that people are very hard to reach and mostly wander to distant areas to look for animal foods, in addition to having poor infrastructure. This finding is assured by its consistency with previous studies conducted in Ethiopia and other African countries [38–40].

Place of residence was also another community-level factor which determines the choice of place of delivery. The odds of delivery at institutions for women residing in urban areas were 5.3 times higher as compared to women living in a rural area. This finding is supported by national surveys in India and urban Bangladesh stating that urban women tend to benefit from increased knowledge and access to maternal health service [19].

## 5. Conclusions

In this study, both the individual- and community-level characteristics were found to have a significant influence on institutional delivery, and we identified spatial clusters of health facility delivery with Afar and Somali region having the lowest utilization rates and Addis Ababa and Tigray having the highest utilization rates. In multivariate multilevel logistic regression analysis, antenatal care visits, household wealth index, maternal education, parity, community level of education, residence, and region were variables that achieved statistically significant association with institutional delivery.

## Figures and Tables

**Table 1:** Sample characteristics of women aged 15–49 who had their most recent live birth in the five years preceding the survey, 2016 EDHS.

Variables	Frequency	Percent
Household wealth index		
Poorest	1,651	21.76
Poorer	1,654	21.79
Middle	1,588	20.93
Richer	1,426	18.80
Richest	1,269	16.72

Place of residence		
Urban	968.85	12.77
Rural	6,620.62	87.23

Mothers age in years		
15–24 years	1,803.98	23.77
25–34 years	2,165.28	28.53
35–49 years	3,620.49	47.70

Maternal educational status		
No education	4,791.00	63.12
Primary education	2,149.63	28.32
Secondary education	419.61	5.53
Higher education	229.51	3.02

Religion		
Orthodox	2,882.10	37.97
Catholic	71.48	0.94
Muslim	1,651.41	21.76
Protestant	2,824.01	37.21
Others	160.74	2.12

Maternal working status		
Not working	5,417.66	71.38
Working	2,172.11	28.62

**Table 2:** Sample characteristics of community level of women aged 15–49 who had their most recent live birth in the five years preceding the survey, 2016 EDHS.

Variables	Community frequency (total: 7,590 women)	Percent (%)
Place of residence		
Urban	968.85	12.77
Rural	6,620.62	87.23
Regions		
Pastoralist	441.26	5.81
Agrarian	6,899.47	90.90
City	249.03	3.28
Community education		
Lower education	3,743.97	49.33
Higher education	3,845.79	50.67
Community poverty		
Lower poverty	4,643.17	61.18
Higher poverty	2,946.59	38.82

**Table 3:** Model comparison using parameters (community-level factors, individual-level factors, and null model for 2016 EDHS).

Variables fixed effect	Model 1	Model 2 AOR (95% CI)	Model 3 AOR (95% CI)	Model 4 AOR (95% CI)
Maternal age				
15–24 years®		1		1
25–34 years		0.83 (0.61–1.12)		1.31 (0.76–2.27)
35–49 years		0.98 (0.68–1.43)		0.91 (0.60–1.39)
Maternal educational				
No education®		1		1
Primary		1.52 (1.21–1.92)		1.40 (1.11–1.77)^*∗*^
Secondary		3.83 (2.49–6.04)		3.25 (2.03–5.19)^*∗*^
Higher		5.41 (2.14–13.4)		3.89 (1.51–10.01)^*∗*^
Wealth index				
Poorest®		1		1
Poorer		1.59 (1.12–2.27)		1.46 (1.03–2.09)^*∗*^
Middle		1.53 (1.11–2.16)		1.39 (0.99–1.95)
Richer		1.60 (1.12–2.40)		1.39 (0.95–2.05)
Richest		4.50 (3.18–7.26)		2.18 (1.39–3.41)^*∗*^
Antenatal care visit				
0®		1		1
1–3		3.84 (2.83–5.21)		3.75 (2.76–5.10)^*∗*^
4 and above		7.02 (5.18–9.51)		6.57 (4.83–8.94)^∗∗^
Birth order				
6 and above®		1		1
1–3		1.44 (0.84–2.48)		1.31 (0.76–2.27)
4–5		0.97 (0.65–1.48)		0.91 (0.60–1.39)
Women working status				
Not working®		1		1
Working		1.12 (0.86–1.44)		1.09 (0.84–1.42)
Parity				
1®		1		1
2–4		0.47 (0.34–0.66)		0.48 (0.34–0.68)^*∗*^
>5		0.56 (0.31–0.99)		0.59 (0.33–1.04)
Relationship to household head				
Head®		1		1
Wife		0.92 (0.66–1.28)		0.96 (0.67–1.37)
Other		1.05 (0.66–1.66)		1.12 (0.69–1.82)
Media exposure				
No®		1		1
Yes		1.13 (0.83–1.55)		1.04 (0.75–1.43)
Distance to healthcare facility				
Not a big problem®		1		1
A big problem		1.32 (1.06–1.64)		1.22 (0.98–1.52)
Regions (community)				
City®			1	1
Pastoralist			0.16 (0.10–0.2)	0.30 (0.19–0.48)^*∗*^
Agrarian			0.31 (0.21–0.5)	0.45 (0.30–0.69)^*∗*^
Residence (community)				
Rural®			1	1
Urban			12.88 (8.27–20)	5.30 (3.10–9.06)^*∗*^
Community poverty				
Lower®			1	1
Higher			0.62 (0.43–0.9)	0.82 (0.56–1.19)
Community education				
Lower®			1	1
Higher			2.95 (2.09–4.1)	1.70 (1.22–2.36)^∗∗^

The symbols are represented as follows: *P* 0.05, *P* 0.01, 1: reference group, ®outcome variables of interest.

**Table 4:** Random effect and model comparison for factors associated with institutional delivery, Ethiopia, 2016.

Parameters	Model 1	Model 2	Model 3	Model 4
ICC	63%	24%	31%	23%
MOR	9.45	2.62	3.2	2.54
PCV	Ref	81.57%	73.52%	82.63%
LLR	−3,660.24	−2,993.19	−3,344.13	−2,911.60

## Data Availability

All result-based data are available within the manuscript, and anyone can access the dataset online from http://www.measuredhs.com.

## Abbreviations

ANC: Antenatal care
CSA: Central Statistical Agency
DHS: Demographic and Health Survey
EDHS: Ethiopia Demographic and Health Survey
EA: Enumeration area
GLMM: Generalized logistic mixed-effect regression model
GIS: Geographic information system
LQAS: Low-quality assistance sampling
ICC: Intraclass correlation coefficient
PCV: Proportional change in variance.

## References

[1] Demographic E. (2006). *Health Survey: Addis Ababa, Ethiopia and Calverton*.

[2] Organization W. H. (2019). *Maternal Mortality: Evidence Brief*.

[3] Kassebaum N. J., Barber R. M., Bhutta Z. A. (2016). Global, regional, and national levels of maternal mortality, 1990–2015: a systematic analysis for the global burden of disease study 2015. *The Lancet*.

[4] Hailu M., Gebremariam A., Alemseged F., Deribe K. (2011). Birth preparedness and complication readiness among pregnant women in Southern Ethiopia. *PLoS One*.

[5] Mekonnen Y., Mekonnen A. (2003). Factors influencing the use of maternal healthcare services in Ethiopia. *Journal of Health, Population and Nutrition*.

[6] Setegn T., Lakew Y., Deribe K. (2016). Geographic variation and factors associated with female genital mutilation among reproductive age women in Ethiopia: a national population based survey. *PLoS One*.

[7] Tarekegn S. M., Lieberman L. S., Giedraitis V. (2014). Determinants of maternal health service utilization in Ethiopia: analysis of the 2011 Ethiopian demographic and health survey. *BMC Pregnancy and Childbirth*.

[8] Blears E. E., Pham N. K., Bauer V. P. (2020). A systematic review and meta-analysis of valued obstetric and gynecologic (OB/GYN) procedures in resource-poor areas. *Surgery Open Science*.

[9] Sayih A. (2014). Factors determining choice of delivery place among women’s of child bearing age in Dega Damot Woreda, West Gojjam Zone, Amhara Regional State, Ethiopia. *Doctoral dissertation*.

[10] Tsegaye B., Ayalew M. (2020). Prevalence and factors associated with antenatal care utilization in Ethiopia: an evidence from demographic health survey 2016. *BMC Pregnancy and Child Birth*.

[11] Colombara D. V., Hernández B., Schaefer A. (2016). Institutional delivery and satisfaction among indigenous and poor women in Guatemala, Mexico, and Panama. *PLoS One*.

[12] Garg R., Shyamsunder D., Singh T., Avtar P. (2010). *Health and Population, Perspectives and Issues Incorporating Nihae Bulletin and the Journal of Population Research*.

[13] Montagu D., Yamey G., Visconti A., Harding A., Yoong J. (2011). Where do poor women in developing countries give birth? a multi-country analysis of demographic and health survey data. *PLoS One*.

[14] Roy M. P., Mohan U., Singh S. K., Singh V. K., Srivastava A. K. (2013). Factors associated with the preference for delivery at the government hospitals in rural areas of Lucknow district in Uttar Pradesh. *Indian Journal of Public Health*.

[15] Exavery A., Kanté A. M., Njozi M. (2014). Access to institutional delivery care and reasons for home delivery in three districts of Tanzania. *International Journal for Equity in Health*.

[16] Amano A., Gebeyehu A., Birhanu Z. (2012). Institutional delivery service utilization in Munisa Woreda, South East Ethiopia: a community based cross-sectional study. *BMC Pregnancy and Childbirth*.

[17] Alemayehu S., Fekadu M., Solomon M. (2012). Institutional delivery service utilization and associated factors among mothers who gave birth in the last 12 months in Sekela District, North West of Ethiopia. A community based cross sectional study. *BMC Pregnancy and Childbirth*.

[18] Mazalale J., Kambala C., Brenner S. (2015). Factors associated with delivery outside a health facility: cross-sectional study in rural Malawi. *Tropical Medicine and International Health*.

[19] Yaya S., Bishwajit G., Ekholuenetale M. (2017). Factors associated with the utilization of institutional delivery services in Bangladesh. *PLoS One*.

[20] Moges A. Y., Yaya T. N. (2017). Determinants of safe delivery service utilization among women of childbearing age in Egela Sub-Woreda, Tigray, Northern Ethiopia. *Science Journal of Public Health*.

[21] Abeje G., Azage M., Setegn T. (2014). Factors associated with Institutional delivery service utilization among mothers in Bahir Dar city administration, Amhara region: a community based cross sectional study. *Reproductive Health*.

[22] Thomas S., Fayter D., Misso K. (2008). Population tobacco control interventions and their effects on social inequalities in smoking: systematic review. *Tobacco Control*.

[23] Agha S., Carton T. W. (2011). Determinants of institutional delivery in rural Jhang, Pakistan. *International Journal for Equity in Health*.

[24] Zepro N. B., Ahmed A. T. (2016). Determinants of institutional delivery service utilization among pastorals of Liben Zone, Somali Regional State, Ethiopia, 2015. *International Journal of Women’s Health*.

[25] Feyissa T. R., Genemo G. A. (2014). Determinants of institutional delivery among childbearing age women in western Ethiopia, 2013. *Unmatched Case Control Study*.

[26] Belay A., Sendo E. (2016). Factors determining choice of delivery place among women of child bearing age in Dega Damot district, North West of Ethiopia: a community based cross-sectional study. *BMC Pregnancy and Childbirth*.

[27] Mehari A. M. (2013). *Levels and Determinants of Use of Institutional Delivery Care Services Among Women of Childbearing Age in Ethiopia: Analysis of EDHS 2000 and 2005 Data*.

[28] Titaley C. R., Hunter C. L., Dibley M. J., Heywood P. (2010). Why do some women still prefer traditional birth attendants and home delivery?: a qualitative study on delivery care services in West Java Province, Indonesia. *BMC Pregnancy and Childbirth*.

[29] Mekonnen M. G., Yalew K. N., Umer J. Y., Melese M. (2012). Determinants of delivery practices among Afar pastoralists of Ethiopia. *The Pan African Medical Jornal*.

[30] Fikre A. A., Demissie M. (2012). Prevalence of institutional delivery and associated factors in Dodota Woreda (district), Oromia regional state, Ethiopia. *Reproductive Health*.

[31] Mubarik S. (2016). Effect of national health insurance holding on the choice of health facility for childbirth in Ghana. *Science Journal of Public Health*.

[32] Tsegay R., Aregay A., Kidanu K., Alemayehu M., Yohannes G. (2017). Determinant factors of home delivery among women in Northern Ethiopia: a case control study. *BMC Public Health*.

[33] Van Blerk L. (2008). Poverty, migration and sex work: youth transitions in Ethiopia. *Area*.

[34] Hodgkin D. (1996). Household characteristics affecting where mothers deliver in rural kenya. *Health Economy*.

[35] Shah R., Rehfuess E. A., Maskey M. K., Fischer R., Bhandari P. B., Delius M. (2015). Factors affecting institutional delivery in rural Chitwan district of Nepal: a community-based cross-sectional study. *BMC Pregnancy and Childbirth*.

[36] brumblay S. K. N., Mbaruku G., Kruk M. E. (2012). Parity and institutional delivery in rural Tanzania: a multilevel analysis and policy implications. *Health Policy and Planning*.

[37] Stephenson R., Baschieri A., Clements S., Hennink M., Madise N. (2006). Contextual influences on the use of health facilities for childbirth in Africa. *American Journal of Public Health*.

[38] Aremu O., Lawoko S., Dalal K. (2012). The influence of individual and contextual socioeconomic status on obstetric care utilization in the democratic republic of Congo: a population-based study. *International Journal of Preventive Medicine*.

[39] Gabrysch S., Cousens S., Cox J., Campbell O. M. R. (2011). The influence of distance and level of care on delivery place in rural Zambia: a study of linked national data in a geographic information system. *PLoS Medicine*.

[40] Utomo B., Sucahya P. K., Utami F. R. (2011). Priorities and realities: addressing the rich-poor gaps in health status and service access in Indonesia. *International Journal for Equity in Health*.

[41] Sisay D., Muche T., Ali H. (2020). *Spatial Distribution and Associated Factors of Institutional Delivery among Reproductive-Age Women (15-49 Years) in Ethiopia: The Case of Ethiopian Demographic and Health Survey 2016 Data*.

